# Evolution of the locomotory system in eels (Teleostei: Elopomorpha)

**DOI:** 10.1186/s12862-016-0728-7

**Published:** 2016-08-11

**Authors:** Cathrin Pfaff, Roberto Zorzin, Jürgen Kriwet

**Affiliations:** 1 Department of Palaeontology, University of Vienna, Faculty of Earth Sciences, Geozentrum, UZA II, Althanstraße 14, 1090 Vienna, Austria; 2 Museo civico di Storia Naturale, Palazzo Pompei, Lungadige Porta Vittoria 9, 37129 Verona, Italy

**Keywords:** Anguilliforms, Bony tendons, Fossil-Lagerstaetten, Functional morphology, Locomotion, Habitat, Lebanon, Pesciara, Monte Bolca, Musculotendinous system

## Abstract

**Background:**

Living anguilliform eels represent a distinct clade of elongated teleostean fishes inhabiting a wide range of habitats. Locomotion of these fishes is highly influenced by the elongated body shape, the anatomy of the vertebral column, and the corresponding soft tissues represented by the musculotendinous system. Up to now, the evolution of axial elongation in eels has been inferred from living taxa only, whereas the reconstruction of evolutionary patterns and functional ecology in extinct eels still is scarce. Rare but excellently preserved fossil eels from the Late Cretaceous and Cenozoic were investigated here to gain a better understanding of locomotory system evolution in anguilliforms and, consequently, their habitat occupations in deep time.

**Results:**

The number of vertebrae in correlation with the body length separates extinct and extant anguilliforms. Even if the phylogenetic signal cannot entirely be excluded, the analyses performed here reveal a continuous shortening of the vertebral column with a simultaneous increase in vertebral numbers in conjunction with short lateral tendons throughout the order. These anatomical changes contradict previous hypotheses based on extant eels solely.

**Conclusions:**

The body curvatures of extant anguilliforms are highly flexible and can be clearly distinguished from extinct species. Anatomical changes of the vertebral column and musculotendinous system through time and between extinct and extant anguilliforms correlate with changes of the body plan and swimming performance and reveal significant shifts in habitat adaptation and thus behaviour. Evolutionary changes in the skeletal system of eels established here also imply that environmental shifts were triggered by abiotic rather than biotic factors (e.g., K/P boundary mass extinction event).

**Electronic supplementary material:**

The online version of this article (doi:10.1186/s12862-016-0728-7) contains supplementary material, which is available to authorized users.

## Background

It is widely appreciated that a close link between phenotype (form), performance (function), fitness, and habitat occupation in extinct and extant vertebrates exists with fishes displaying the largest morphological disparity [1, 2]. One of the most important manifestations of performance is locomotion, which differs between land-living and aquatic vertebrates mainly due to gravity differences. In water, forward propulsion is accomplished either by the action of fins or by the activity of fins and body. The most common swimming mode in a variety of ecologically and morphologically divergent fishes and aquatic tetrapods is axial-based undulatory locomotion. This type of locomotion generally is subdivided into a range of types such as thunniform, subcarangiform, carangiform, and anguilliform swimming, with the latter being considered one of the extremes along the locomotion continuum [3, 4].

An elongated and slender eel-like body form with a very reduced caudal peduncle and serpentine-like waves of lateral body deflections characterizes anguilliform swimmers [5, 6]. Additionally, their myomers are short, which indicate a high manoeuvrability of the trunk and consequently is assumed to represent an adaptation to structurally complex habitats such as reefs [2, 7–10]. Generally, it is supposed that the length of the axial skeleton increased during anguilliform evolution as a result of various anatomical changes [6, 10–14]. However, these assumptions are based exclusively on analyses of extant anguilliforms.

The swimming capabilities of fishes are highly influenced by the anatomy of the musculotendinous system, which is constant within gnathostomes and considered homologous in teleostean fishes such as, e.g., acanthomorphs ([15]; but see [16] for a different view). The musculotendinous system consists of sheet-like connective tissue (myosepta) that divides the muscles into separate myomeres. The horizontal septum separates the myomeres into dorsal and ventral portions. The length of the myosepta correlates with the length of the lateral tendon and are either isochronous throughout the trunk, characterizing the anguilliform swimming mode [7, 17], or are elongated in the posterior body region as in thunniform and carangiform swimmers [18]. The attachment of the musculotendinous system to the vertebral column influences the flexibility of the trunk and correlates with locomotion performance that can be deduced by studying the point of insertion and the course of its different components [7, 9, 17, 19, 20]. This has the potential to provide important information about the evolutionary ecology of locomotion patterns and habitat occupations of extant fishes [7, 9, 19].

However, macroevolutionary patterns and functional-morphological adaptations of extinct fishes such as anguilliforms still have not been assessed up to now, because specimens with completely and exceptionally preserved soft-tissues of the musculotendinous system are rare in the fossil record. Different function-related morphological features such as the lower jaw and eye-diameter [21, 22] but also morphometric data [23] have been used instead for inferring the ecological role of extinct fishes so far. Moreover, all available analyses of evolutionary patterns and evolutionary biology such as functionalities and related habitat adaptations of extinct fishes are restricted predominantly to acanthomorph teleosts without considering anguilliforms (e.g., [24, 25]). Nonetheless, fossil fishes add significant deep-time evolutionary aspects to analyses based on living taxa and have the potential to identify trait changes by polarizing morphological characters and identifying adaptive patterns.

Well-preserved anguilliforms displaying soft tissue structures associated with the axial skeleton occasionally occur in conservation Lagerstaetten of Late Cretaceous, Palaeogene, and Cenozoic age. These specimens that form the focus of this study include both basal and derived forms, which enable us to reconstruct evolutionary patterns in the vertebral column and the musculotendinous system. In the following, the fossil localities that yielded these specimens are summarized.

The stratigraphically oldest known anguilliforms (†*Abisaadia hakelensis*, †*Anguillavus mazeni*, †*Anguillavus quadripinnis*, †*Luenchelys minimus*, †*Urenchelys germanum*) originated from open-marine platform deposits of the Sannine Limestone, which is of Cenomanian age (99-98 Ma) and were recovered from three localities in Lebanon (Hajula, Hakel, Namoura) [26, 27]. Deposition of the fossiliferous sediments occurred either in an outer (Hadjula, Hakel) or upper carbonate platform setting (Namoura). These anguilliforms are important because they represent members of the stem-group of anguilliforms [28–30] (Fig. 2) and document the earliest record of anguilliform body plans. The musculotendinous system is completely preserved only in the holotype of †*Luenchelys minimus,* but, unfortunately, not preserved in all other studied Cenomanian anguilliforms.

Palaeogene anguilliforms examined here are from the world-famous fossil locality of Pesciara in northern Italy, which is of Middle Eocene age close to the Ypresian/Lutetian boundary (ca. 50 Ma). This is one of the most productive marine fish conservation Lagerstaetten of Eocene age. The most striking feature of this fish assemblage is the tremendous preservation of specimens, which generally are completely articulated and often display soft tissues including pigmentation patterns (e.g., †*Paranguilla tigrina*). The presence of abundant larvae and also large, fully-grown adults of different clades indicates that taphonomic biases are negligible [31]. Fishes were deposited under anoxic conditions in coastal basins, which were surrounded by hard-grounds [32]. Reefeal structures, however, are not known from the vicinity indicating that these fossiliferous limestones most likely were deposited in basin structures or on a wide shelf. Nevertheless, the fish assemblage is of crucial importance because it heralds the first appearance of several lineages of fishes, which are important groups occurring today in coral reefs [23, 31, 33, 34]. Additionally, it generally is assumed that fishes in the Pesciara assemblage also had similar ecologies as their living counterparts [22].

The only analysed Neogene anguilliform comes from the fossil locality of Oehningen in Baden-Württemberg, south-eastern Germany, which is one of the classic fossil sites of central Europe. Fossiliferous deposits were accumulated in a maar during the Miocene Upper Freshwater Molasse (ca. 13 Ma) [35]. Thus, this assemblage represents a mixture of freshwater and terrestrial organisms including abundant fishes and the oldest figured specimen of an anguilliform [36] that was identified as a species of freshwater anguillids, *Anguilla elegans* [37]. The only known Eocene anguillid, †*Anguilla ignota* [38] was not included in this study, because no detailed information about its axial skeleton could be obtained.

Here, we intend to identify adaptations in the locomotor apparatus as expressed by the musculotendinous system in exceptionally preserved Cretaceous and Cenozoic anguilliforms to identify evolutionary patterns in a phylogenetic framework and predict habitat adaptations with functional ecology in extinct eels.

## Methods

**Fig. 1:**
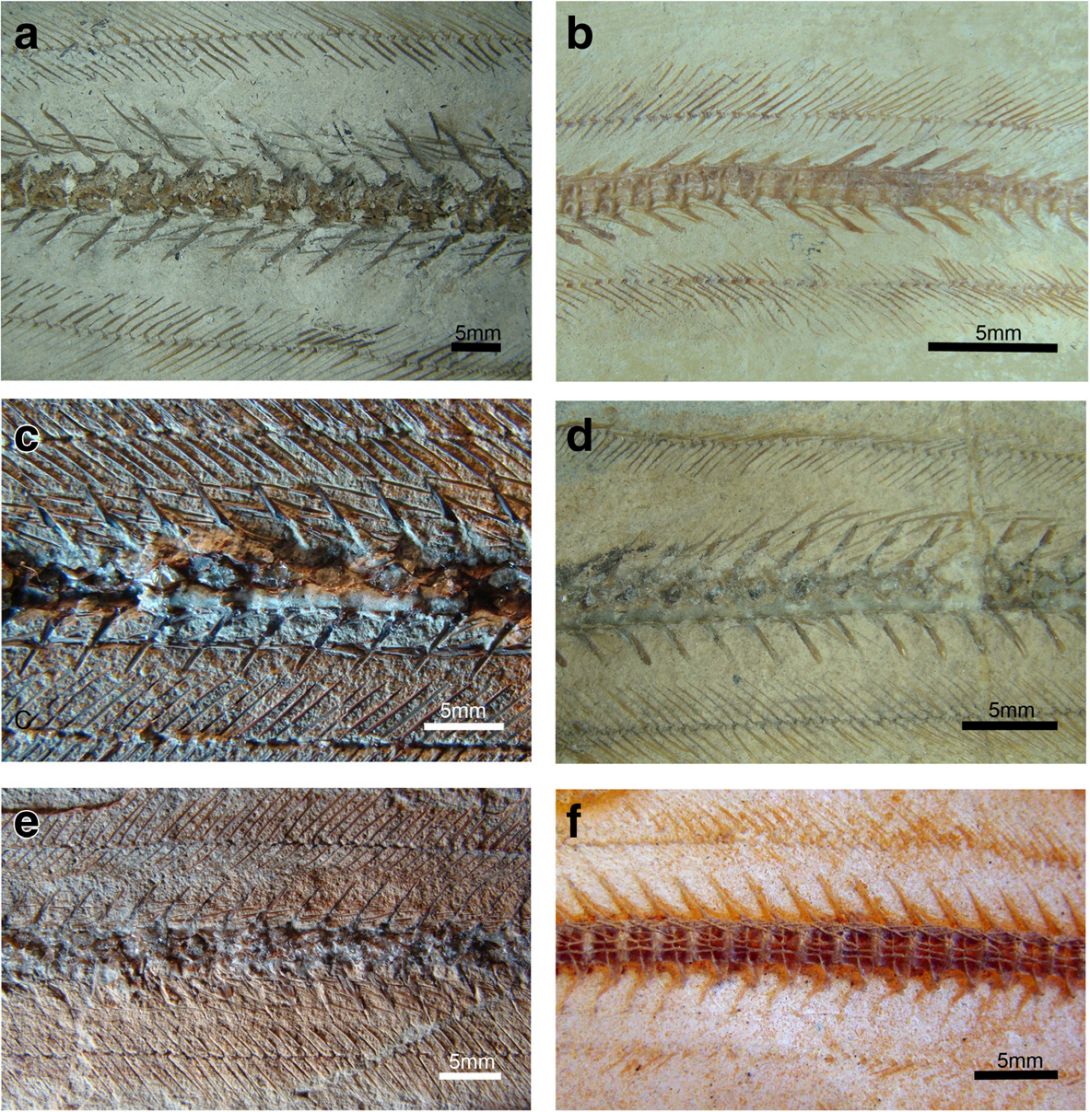
Figures depicting details of the caudal body in fossil anguilliforms. **a** †*Anguilla elegans* (NHMUK 42769); **b** †*Luenchelys minimus* (NHMUK P.62692.a); **c** †*Bolcyrus formosissimus* (MSNVR T.468); **d** †*Voltaconger latispinus* (NHMUK P.1889); **e** †*Anguilloides branchiostegalis* (MSNVR VII.A.18); **f** †*Anguilloides branchiostegalis* (NHMUK P.3876)

**Fig. 2:**
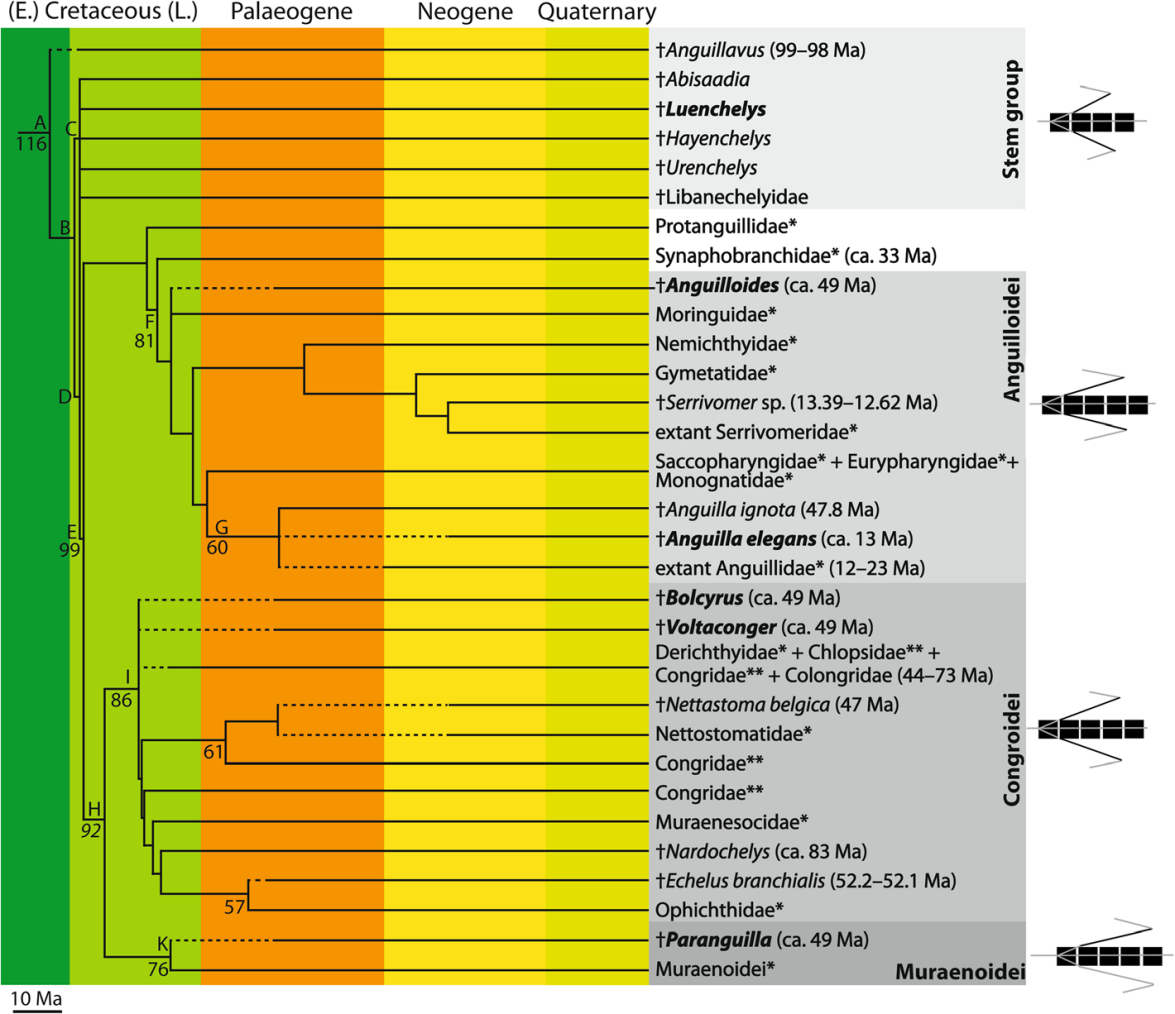
Calibrated composite phylogeny of extant and extinct Anguilliformes depicting the relationships of fossil and extant anguilliforms, divergence ages and evolutionary changes in the musculotendinous system (on the right) showing the length of the tendons in relation of vertebral centra. Daggers (†) preceding names indicate extinct taxa. Names in bold are extinct taxa examined in this study. Solid lines of extinct taxa indicate the stratigraphic age of fossils; dashed lines represent ghost-lineages as inferred from their systematic position. Letters above lines depict monophyletic groups referred to below. Numbers below lines are minimum divergence estimates based on molecular clock approaches [27]. An asterisk following terminal taxa names indicates living clades, two asterisks depict paraphyletic groupings [27]. Ages of terminal taxa are assembled from various sources (e.g., [27]). Italicised numbers are divergence estimated inferred from the stratigraphic distribution of taxa (node B) or calculated as mean between two bracketing estimates (divergence between ‘congroids’ and muraenoids). For detailed explanations see text

**Fig. 3:**
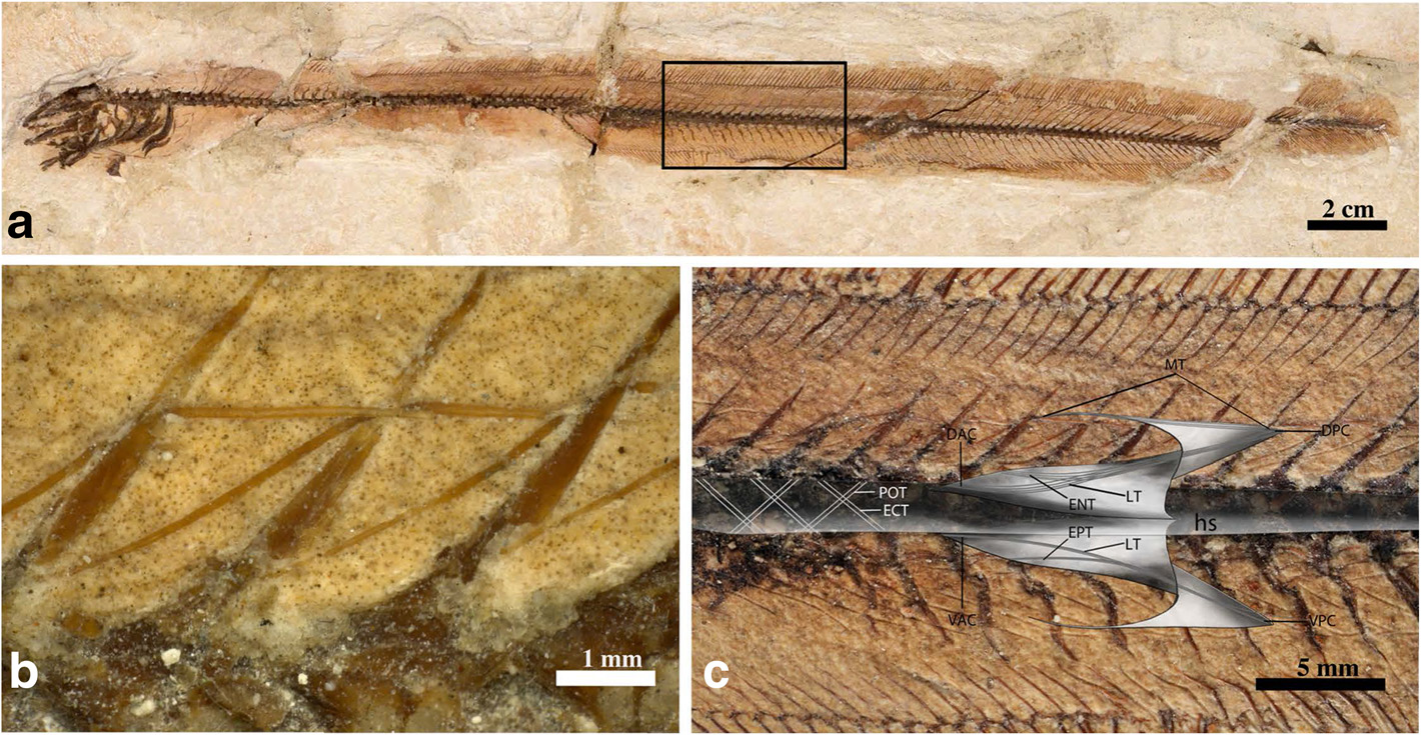
Osteology and musculotendinous system of †*Anguilloides branchiostegalis*. **a** NMW A.3319, image, **b** and detail **c** reconstruction of a three-dimensional myosepta and the horizontal septum (anterior to the left). DAC – dorsal anterior cone; ECT - epicentral tendon; ENT – epineural tendon; EPT – epipleural tendon; hs - horizontal septum; LT - lateral tendon; MT – myorhabdoid tendon; POT - posterior oblique tendon; VAC – ventral anterior cone; VPC - ventral posterior cone

For investigating the evolution of the locomotion in anguilliforms, we analysed 39 fossil specimens housed in the collections of the natural history museums of Verona (Italy), Vienna (Austria), and London (UK) in detail of which 15 specimens display preserved musculotendinous systems in the posterior body (Figs. 1 and 3, Additional file 1: Figure S1, Additional file 2: Table S1, Additional file 3: Note 1). The phylogenetic framework and procedure for phylogenetic tree building of the investigated taxa are depicted in Fig. 2.

### Composite phylogeny of extant and extinct Anguilliformes

A cladistic analysis of anguilliforms employing robust phylogenetic approaches based on morphological traits and comprising fossil and extant taxa, which would necessitate a major revision of anguilliform taxa involved is beyond the scope of this study because we were not able to study all necessary specimens (especially those of living clades) to establish character sets for phylogenetic analyses. We did not employ supertree or supermatrix approaches because the available source phylogenies generally consider only taxonomically restricted clades lacking overlapping leaf sets. Additionally, we did not use previously published divergence estimates (e.g., [39–41]).

Conversely, we compiled a composite tree of extinct and extant anguilliforms from many independent studies for evaluating the evolutionary changes in the musculotendinous system (Fig. 2). Our composite phylogeny is based on a backbone cladogram of living anguilliforms, which represents the most comprehensive, recent molecular analysis [27]. The sister group relationship between Anguilliformes and Notacanthiformes within Elopomorpha is well established (e.g., [27, 41]). A mean age estimate for divergence between both of 123.5 Ma and a 95 % confidence interval of 152.7 Ma was previously provided [41].

### Body shape analyses

Detailed measurements of extinct specimens were conducted with tpsUtil v.1.58 and tpsDig v.1.40 and combined with data of extant anguilliforms ([13]; Additional file 2: Table S1). The relationship between body length and number of vertebrae was analysed with linear regression analyses conducted by SPSS 20.0. (IBM, Armonk, USA).

**Fig. 4:**
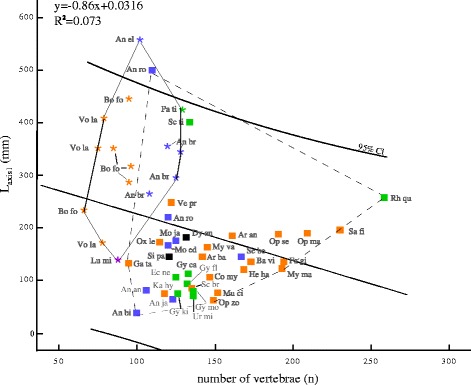
Regression analysis of total body length in mm (L_axis1_) and number of vertebrae (n). asterisks, fossil anguilliforms; squares, living anguilliforms; 95 % confidence interval (CI). violet: stem group; black: Synaphobranchidae; blue: Anguilloidei; orange: Congroidei; green: Muraenoidei; Abbreviations of the taxa are as follows: An el, †*Anguilla elegans;* An br, †*Anguilloides branchiostegalis*; An an, *Anguilla anguilla*; An bi; *Anguilla bicolor*; An ja, *Anguilla japonica*; An ro, *Anguilla rostrata*; Ar an, *Ariosoma balearicum*; Ba vi, *Bathyuroconger vicinus*; Bo fo, †*Bolycyrus formosissimus*; Co my, *Conger myriaster*; Dy an, *Dysomma anguillare*; Ec ne, *Echidna nebulosa*; Fa gi, *Facciolella gilbertii*; Ga ta, *Gavialiceps taeniola*; Gy ca, *Gymnothorax castaneus*; Gy fl, *Gymnothorax flavimarginatus*; Gy ki, *Gymnothorax kidako*; Gy mo, *Gymnothorax moringa*; He ha, *Heteroconger hassi*; Ka hy, *Kaupichthys hyoporides*; Lu mi, †*Luenchelys minimus*; Mo ed, *Moringua edwardsi*; Mo ja, *Moringua javanica*; Mu ci, *Muraenosox cinereus*; My ma, *Myrichthys magnificus*; My va, *Myrophis vafer*; Op ma, *Ophichthus maculosus*; Op se, *Ophichthus serpentinus*; Op zo, *Ophichthus zophochir*; Ox le, *Oxyconger leptognathus*; Pa ti, †*Paranguilla tigrina*; Rh qu, *Rhinomuraena quaesita*; Sa fi, *Saurenchelys fierasfer*; Sc br, *Scolecenchelys breviceps*; Sc ti, *Scuticaria tigrina*; Se be, *Serrivomer beanii*; Si pa, *Simenchelys parasitica*; Ur mi, *Uropterygius micropterus*; Ve pr, *Venefica proboscidea*; Vo la; †*Voltaconger latispinus*

**Fig. 5:**
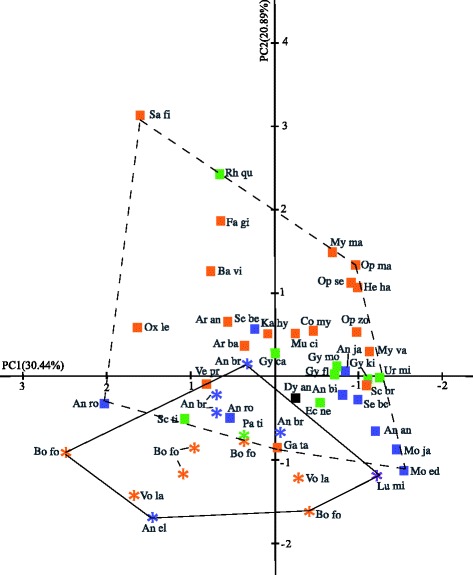
Shape differences of the body form of fossil and living anguilliforms calculated with PCA. 51.33 % of the among group variance with a set of eight variables. Abbreviations see Fig. 4

The ‘vertebrate shape index’ (VSI) is employed here, which represents a metric of the body shape and, therefore, describes the shape diversity in vertebrates [13]. It quantifies the shape of the body by the computation of different morphometric indices of the investigated fishes. Comparison of VSI among extinct and extant anguilliforms was scrutinized with principal component analyses (Figs. 4 and 5, Additional file 2: Table S1) using the following formula [13]:

$$ \mathrm{V}\mathrm{S}\mathrm{I} = \left({\mathrm{L}}_{\mathrm{axis}1}/{\mathrm{L}}_{\mathrm{axis}2}\right) + \left({\mathrm{L}}_{\mathrm{head}\ \mathrm{in}\ \mathrm{vertebrae}}\mathrm{x}\ {\mathrm{AR}}_{\mathrm{head}}\right) + \left({\mathrm{N}}_{\mathrm{PCV}}\mathrm{x}\ {\mathrm{AR}}_{\mathrm{PCV}}\right) + \left({\mathrm{N}}_{\mathrm{CV}}\mathrm{x}\ {\mathrm{AR}}_{\mathrm{CV}}\right). $$

Abbreviations of the VSI index are as follows:

AR_CV_ - mean aspect ratio of the length of three selected caudal vertebrae to their height in the dimension of L_axis2_; AR_head_ - ratio of head length to its length in the dimension of L_axis2;_ AR_PCV_ - mean aspect ratio of three selected precaudal vertebrae to their height in the dimension of L_axis2_; L_axis1_ - standard length; L_axis2_ - length of secondary body axis (maximum body depth or width; in extinct anguilliforms body depth is used); L_head in vertebrae_ - head length quantified as the number of vertebrae spanning the anterio-posterior length of the head; N_PCV_ - number of precaudal vertebrae; N_CV_ - number of caudal vertebrae; VSI - vertebrate shape index.

### Fossilized musculotendinous system

Structural features of the postcranial body including secondarily ossified soft tissues (myoseptal tendons) were studied in extinct anguilliforms by light microscopy and visualized with polarized light (VHX 1000; Keyence, Osaka, Japan) (Figs. 1 and 3, Additional file 2: Table S1, Additional file 3: Note 1). The morphological terminology for myosepta and the definition of the axial position in anguilliform swimmers employed here follows previous studies (e.g., [7, 9, 15, 42]). The number of traversed vertebrae by the epineural (ENB) and epipleural bones (EPB) provides a fairly accurate estimate of the length of the lateral tendon. The total length of the lateral tendon and, therefore of the myoseptum, is calculated by adding one additional segment (*N*) based on comparative investigations in extant eels [7] and the corresponding distance between the lateral tendon and ENB, respectively EPB (Figs. 1 and 3, Additional file 2: Table S1). Detailed measurements of ENB and EPB lengths were taken in millimetres with a stereomicroscope (Nikon SMZ 1500, Chiyoda, Japan) using a 2 mm standard scale bar slide with 20 subdivisions ([9]; Additional file 2: Table S1). Resulting myoseptal length is expressed in relation to total body length and compared to previously published data [7, 9, 17]. We established the ancestral states of myoseptal length with Mesquite 3.02 [43] by using the ancestral state module and the parsimony method without estimated time divergences (Additional file 1: Figure S1).

The influence of the phylogenetic signal in the principal component analysis was determined with Bloomberg’s K-value and Pagel’s λ. The analyses were carried out with Mesquite 3.02 [43], R-Studio v. 0.99.484 [44], and the packages ape 3.4 [45, 46], phylobase v.0.8.2 [47], geiger v.2.0.6 [48] and phytools v.0.5-10 [49] following previous procedures [46]. For a detailed list of results see Additional file 2: Table S1.

## Results

Detailed measurements of the investigated specimens are listed in the electronic supplementary material (Additional file 2: Table S1).

### Composite phylogeny of extant and extinct Anguilliformes

The extinct anguilliforms, †*Anguillavus* and †*Hayenchelys* were identified as stem group members in a phylogenetic analysis [28] and are placed accordingly at the base of the tree. Extinct taxa such as †*Abisaadia* and †*Libanchelys,* that have not been included in any phylogenetic analysis up to now, were inserted manually by us at the conservative position of minimum assumption based on published information [26, 29, 30, 39, 50, 51]. The interrelationships of most stem-group representatives or even crown anguilliforms (e.g., *Anguilla*) consequently are unresolved displaying extensive polytomies. This, however, is not disadvantageous since we intend to identify evolutionary trends across clades rather than within smaller monophyletic units. The resulting tree was calibrated using the oldest occurrence of taxa and/or clades as extension ranges (Fig. 2). In doing this, we did not distinguish between actual stratigraphic ranges based on fossil occurrences and ghost lineages, since this is of no relevance here and does not influence our results. Nevertheless, origination and divergence estimates used here represent hard minimum rather than soft maximum age constraints.

The approach provides some new information about the interrelationships of extinct and extant anguilliforms even though it does not follow strict cladistics principles. Therefore, we provide a summary of the major results (Fig. 2), which are important for our evolutionary deductions. Node A depicts the sister group relationships between the most basal anguilliform, †*Anguillavus* from the Cenomanian (99-98 Ma) and all remaining anguilliforms. †*Anguillavus* is represented by two, probably synonymous, species [26]. Two other possible anguilliforms, †*Enchelion* and †*Enchelurus*, are known from the Cenomanian but are excluded here because of their unknown systematic status (see also [26]). The basal position of †*Anguillavus* is supported, *inter alia*, by the presence of reduced but still present pelvic fins and girdle [29, 51]. All other anguillforms lack the pelvic girdle. Divergence between †*Anguillavus* and all other remaining anguilliforms occurred at ca. 116 Ma [27] indicating that anguilliforms might have originated in the Aptian (Early Cretaceous).

Above node B an unresolved clade including †*Abisaadia*, †*Luenchelys*, †*Hayenchelys*, †*Urenchelys*, †Libanechelyidae, and crown-group anguilliforms (node D) form sister groups. Divergence is estimated here at ca. 116-99 Ma based on fossil occurrences. The clade comprising [†*Abisaadia* + †*Luenchelys* + †*Hayenchelys* + †*Urenchelys*] and †Libanechelyidae are thus supported here as stem-group anguilliforms. Nineteen characters support the monophyly of a clade above node C [51]. †*Abisaadia* might represent the most basal member of clade C because it still occasionally shows remains of the pelvic girdle and fins [29, 51]. †Libanechelyidae seemingly is the most advanced stem-group representative occupying an intermediate position between more basal stem group and crown-group members and is assumed to represent the sister taxon of crown anguilliforms [51].

Node E defines a monophyletic clade comprising all crown-group anguilliforms; divergence of crown-group anguilliforms is dated at ca. 99 Ma based on molecular data [27]. This correlates more or less with the oldest fossil occurrences of stem anguilliforms. The oldest fossil remains of clade E are known from the Santonian of Italy [29] and the Campanian-Maastrichtian of North America and Italy, respectively ranging from 84.7 – 74.5 Ma [52, 53]. This indicates that the origin of the total and crown clade as well as diversification of stem and basal crown members occurred between 116 and 74 Ma corresponding to a time when the climate was very warm and the supercontinent Pangea continued to break up resulting in the establishment of new near-shore habitats.

Node F represents the common ancestor of Anguilloidei (81 Ma). †*Anguilloides* from the Eocene of Italy (ca. 49 Ma) is a member of this clade but of uncertain systematic position. Within Anguilloidei, the family Anguillidae (node G) includes two fossil taxa, †*Anguilla ignota* from the Eocene (47.8 Ma) and †*Anguilla elegans* from the Miocene (ca. 13 Ma) of Germany, which can be considered as stem-group members of this clade. Nevertheless, all three terminal taxa are arranged in a polytomy here lacking detailed morphological trait analyses. †*Anguilla ignota* is known from maar deposits indicating that freshwater adaptation occurred early in the evolutionary history of anguillids. The origin of the total and crown groups is dated at ca. 60 Ma.

Congroid anguilliforms represent the sister group to muraenoids (node H) and a diverse group above node I. Their origin as inferred from bracketing divergence dates is estimated at 92 Ma. The genera †*Bolcyrus* and †*Voltaconger*, both known from the Eocene of Italy (ca. 49 Ma) are considered here to represent members of congroids with an uncertain relationship to other members. Most likely, they represent stem group members pending further phylogenetic analyses. The stem age for congroids is 86 Ma, which is in good accordance with the oldest known congroid, †*Nardoechelys*, from the Campanian-Maastrichtian of Italy [51].

The Eocene anguilliform †*Paranguilla* resembles that of muraenoids and places this taxon on the stem lineage of Muraenoidei [50]. The origin of the total-group Muraenoidei (node K) inferred from the fossil record dates back to 76 Ma, which indicates a major gap in our knowledge about their evolutionary history since divergence between congroids and muraenoids dates at ca. 92 Ma.

### Analysis of body shape

Two morphospaces occupied by the investigated families of extant and extinct anguilliforms are evident (Fig. 4). The correlation analysis, which compares body length (L_axis1_) with the total number of vertebrae (n) revealed two distinct morphospaces, which are characterized by members of extant and extinct anguilliforms, respectively. The morphospace of extant taxa is larger than the one of fossil taxa with an overlapping region comprising †*Anguilloides branchiostegalis* and *Anguilla rostrata* (Fig. 4). Throughout all investigated clades, extinct taxa have fewer vertebrae with shorter vertebral centra than extant ones. The highest number of vertebrae is found in the muraenoid *Rhinomuraena quaesita*, the congroid *Saurenchelys fierasfer*, and the anguilloidei *Scolenchelys breviceps*. The longest species of all investigated specimens are represented by †*Anguilla elegans* and *Anguilla rostrata.*

In the second analysis, the ‘vertebral shape index’ (VSI) is calculated to quantify body shapes in vertebrates [13]. Extinct and extant taxa occupy two different morphospaces with extant taxa having a larger morphospace than extinct species (Fig. 5). Additionally, an overlapping area of the morphospaces is identifiable containing the investigated specimens of †*Anguilloides branchiostegalis*, †*Paranguilla tigrina*, *Anguilla rostrata*, *Scuticaria tigrina,* and *Gavialiceps taeniola*. The precise loadings of the components of the principal component analyses are provided in the electronic supplementary material (Additional file 2: Table S1). The first component (PC1) correlates positively with the length and width of the body, the length of the skull, and the ratio between skull length and skull width. It correlates negatively with the number of precaudal and caudal vertebrae, and the ratio of length and width of the related vertebrae. The second principal component (PC2) correlates positively with the number of precaudal and caudal vertebrae, the length of the head in relation to the length of the individual vertebral centra, the ratio of the length and width of caudal vertebrae, and the ratio of head length and head width. This component correlates negatively with the length and width of the body, and the ratio of the width and length of the precaudal vertebrae. The third principal component (PC3) correlates positively with the total body length, the number of precaudal vertebrae, the ratio between the length and width of precaudal and caudal vertebrae, and the ratio of the width and length of the head.

### Anatomy of the musculotendinous system in extinct anguilliforms

The ‘W’-shape of the myosepta in extinct anguilliform fishes is identified by the fossilized epineural bone (ENB) and epipleural bone (EPB), and, in one specimen, by the dorsally positioned myorhabdoid tendon (MT) (Figs. 1 and 3). In all investigated species, ENB and EPB are identified at different positions of the body, inclined posterodorsally and posteroventrally in the backward flexure of the corresponding myoseptum, and mostly can be traced to the posterior-most caudal region (Figs. 1 and 3, Additional file 2: Table S1). The length of ENB and EPB indicate the attachment line of the myosepta on the vertebral axis. The length of the lateral tendon, which represents the length of the myosepta, is inferred from the number of traversed vertebrae by ENB or ENP, and by adding one vertebra, which is spanned by DAC and VAC as seen in the extant specimens of *Anguilla rostrata* [7]. Attachment lines and length of myosepta of all investigated specimens are found in the supplementary material (Additional file 2: Table S1).

In stem group anguilliforms, represented here by †*Luenchelys minimus,* the attachment line of the myosepta traverses two vertebrae (*N* + 1). Adding the length of one additional vertebra corresponding to the anterior dorsal and anterior ventral cone, respectively, the length of the lateral tendon can be assumed to have traversed three vertebrae (Additional file 2: Table S1).

In †*Anguilloides branchiostegalis*, the attachment line of the myosepta attaches on the dorsal margin of the vertebral centrum *N* and crosses posterodorsal and posteroventral two subsequent vertebrae (*N* + 2). Adding the additional traversed vertebra of the lateral tendon, four vertebrae represent the length of the myosepta. Dorsally arranged MTs are preserved between 19–46 %TL. The ossified tendons of the horizontal septum can be identified as posterior oblique tendons (POTs), whereas epicentral bones (ECB) are not preserved.

In crown anguilloids represented here by †*Anguilla elegans*, ENB and ENP are traversing one to two vertebrae (*N* + 1/2). Adding the additional crossed vertebra, the length of the lateral tendon is represented by four vertebrae.

In the investigated fossil congroids, †*Bolcyrus formosissimus* and †*Voltaconger latispinus,* ENB attaches epaxially on the dorsal margin of the vertebral centrum *N* and continues posterodorsally across three subsequent vertebrae (*N* + 2) disappearing in the posterior body region between 89–97 %TL. Hypaxially, the EPB proceeds posteroventrally and at least traverses two additional vertebrae (*N* +1) but not more than three segments (*N* + 2) and disappears in the last 3–10 %TL. It is possible to infer a total length of four vertebrae hypaxially based on the epaxially and hypaxially symmetrical anatomy of the musculotendinous system and the additionally traversed vertebra of the lateral tendon.

The length of ENB and EPB decreases due to reduced length in posterior vertebrae. The dorsally positioned MT appears between 56–64 %TL and disappears at 85%TL. The hypaxial MT is only preserved in a single investigated specimen.

†*Paranguilla tigrina*, which is assumed to represent a basal muraenoid and which also displays preserved soft tissues in the posterior body portion, displays the longest lateral tendons of all investigated extinct taxa (Additional file 2: Table S1). The total length of the lateral tendon of this extinct taxon equals 4.5 vertebrae as indicated by the epaxial myosepta, which traverses *N* + 2.5 plus the additionally included vertebrae. Thus, basal muraenoids seemingly have the longest lateral tendon of anguilliform fishes. Hypaxially, no bony tendons are preserved.

## Discussion

Macroevolutionary patterns and the evolutionary biology of anguilliforms within a stratigraphic and phylogenetic framework with additional analyses of exceptionally preserved anguilliforms can be reconstructed and enable us to identify plesiomorphic and homoplastic traits and, additionally, provide soft minimum age constraints for evolutionary events [54]. However, evolutionary studies of axial elongation in anguilliforms have focussed on extant species only so far [6, 11, 13]. It is generally assumed that extinct fishes had similar ecologies as their living counterparts, which consequently can be reconstructed [55]. A major result of our study is that extant and extinct anguilliforms can be separated in the anatomy of their vertebral column and the musculotendinous system.

### Vertebral column

The length of the vertebral column and the corresponding number of vertebrae changes in the evolution of anguilliforms. Few vertebrae with long vertebral centra characterize extinct species in contrast to more and shorter vertebral centra and additional vertebral joints and a comparably shortened trunk in living taxa (Fig. 4). This strongly contradicts previous studies focusing on the effect of ‘pleomerism’ in extant fishes, concluding that larger species have more vertebrae and a positive correlation between vertebral number and maximum body length [12, 56, 57]. However, correlating body length with vertebral numbers already was considered as an inaccurate approach [58] and also is not supported by the overall evolutionary patterns seen in extinct and extant anguilliforms here.

The body shape of anguilliforms can be clearly separated from other groups of vertebrates [13]. To elucidate the anatomical difference of extinct and extant anguilliforms, a principal component analysis was conducted here (Fig. 5). The anatomical distinction between extinct and extant anguilliform is mainly caused by the second principal component (PC2), which has the highest loading on the number of caudal and precaudal vertebrae. This differentiation also is seen in the regression analysis of the number of vertebrae with fewer vertebrae in extinct than in extant taxa (Fig. 4). However, the investigated families of anguilliforms cannot be separated clearly from one another caused by the outliers of †*Paranguilla tigrina, Rhinomuraena questita,* and *Scuticaria tigrina* (Figs. 4 and 5). The reasons for these prominent positions remain ambiguous for the moment but might be related to special adaptations, like in the ribbon eel, *Rhinomuraena questita*, a very slender muraenoid living in burrows of sandy or stony areas adjacent to coral reefs, which are stabilized with a very effective adhesive mucus that is secreted by the fish [59].

In fishes, the number of vertebrae is highly influenced by the size of the species, the body shape, the swimming mode, the variability across taxa and populations but also by the phylogenetic position and ontogeny [60]. The vertebral number is fixed early in the ontogeny of fishes [41, 56, 61, 62, 63] and, therefore, changes must occur during the embryonic development or soon after hatching [63]. The vertebrate segmentation clock and the corresponding oscillator mechanism mainly influence genetically the development of the vertebral column in vertebrates (Notch, Wnt, and Fgf pathways) (e.g., [64–67]). Changes of the environment (e.g., salinity) and climate (e.g., temperature) might influence these mechanisms and thus might correlate with anatomical changes in the vertebral column during the evolution of anguilliforms with shifts in swimming performance and in habitat occupation. However, variation in vertebral numbers usually occurs only within a narrow range [63, 68].

### Musculotendinous system

The distribution and length of the tendons of the musculotendinous system form parts of the force transmission in the fish body [7, 18, 19] and signify ecological and functional-morphological signals of the trunk [9, 17]. The musculotendinous system of extinct eels changes from stem representatives to crown anguilliforms in all aspects of the anatomy of soft tissue. The length of the myosepta and the corresponding attachment line prolongate epaxially and hypaxially in crown species, whereas in the examined stem anguilliform, †*Luenchelys minimus*, the myosepta are comparably shorter (Fig. 2; Additional file 2: Table S1). However, additional comparative analyses of extant anguilliforms are necessary to understand whether these anatomical differences of the musculotendinous system result in distinctive locomotory modes in extinct species.

### Evolutionary and palaeoecological aspects

Overall, shifts in swimming performance and thus in habitat occupation caused by changes of the vertebral column and musculotendinous system in extinct anguilliforms may be explained by the evolution of crown anguilliforms, which is characterized by multiple divergence events between 86 and 76 Ma resulting in the establishment of the five major clades based on the calibrated composite phylogeny here (Fig. 2). Accordingly, the split between congroids and muraenoids occurred ca. 92 Ma and the split between anguilloids and the remaining anguilliforms ca. 99 Ma. Even if we can not exclude the phylogenetic signal in the anatomy of the vertebral column entirely, anatomical changes of the vertebral column may be caused by abiotic factors of the environment, e.g. salinity and/or temperature [60] rather than phylogenetic relationships.

Freshwater adaptation in eels seemingly occurred after the K/P boundary event, latest in the Eocene, whereas adaptation to coral reefs seemingly occurred later (probably not until the Neogene) as suggested by fossil occurrences and interrelationships of eels (Fig. 2). Thus, we hypothesize that the famous fossil fish fauna from the Eocene of Pesciara, which includes abundant eels represents an open-water assemblage.

## Conclusion

Here, we present analyses of the vertebral column and associated soft tissue structures in extinct and extant anguilliform eels. We demonstrate that the anatomy of the vertebral column and musculotendinous system in fossil anguilloids and congroids differs considerably from extant anguilloid fishes. Differences of precaudal and caudal vertebral numbers and body length between extant and extinct taxa indicate a functional-morphological signal in the trunk in anguilliforms and therefore, differences in habitat occupation in the course of their evolutionary history. Thus, it is possible to reconstruct macroevolutionary patterns and habitat shift of anguilloids based on these structures. It is obvious, that the vertebral column continuously became shorter while concurrently an increase in the number of vertebral centra in conjunction with short lateral tendons is recognizable. These alterations indicate changes in locomotor and swimming performance pointing to migrating behavioural shifts probably from open to complex habitats during the evolution of anguilliform fishes. A phylogenetic influence in the anatomy of the vertebral column and musculotendinous system could not be entirely excluded, resulting in these morphological changes. However, abiotic factors in the aftermath of the K/P boundary mass extinction event evidently were the driving forces for these modifications.

## Additional files

Additional file 1: Figure S1. Reconstruction of ancestral state of distinct parameters of the musculotendinous system and body shape. (PDF 285 kb) Additional file 2: Table S1. Measurements and statistic analyses of the vertebral column and musculotendinous system of extant and extinct anguilliforms. (XLS 109 kb) Additional file 3: Note 1. A. Supplementary information of the investigated specimens. B. Stratigraphic and geographic distribution of analysed fossil taxa. (DOC 26 kb)
