# Supplementary figures and images for: Evolution of the locomotory system in eels (Teleostei: Elopomorpha)
Source: BMC Evol Biol. 2016 Aug 11;16:159. doi: 10.1186/s12862-016-0728-7 (PMC4981956; doi:10.1186/s12862-016-0728-7)

Figure S1: Ancestral state reconstruction

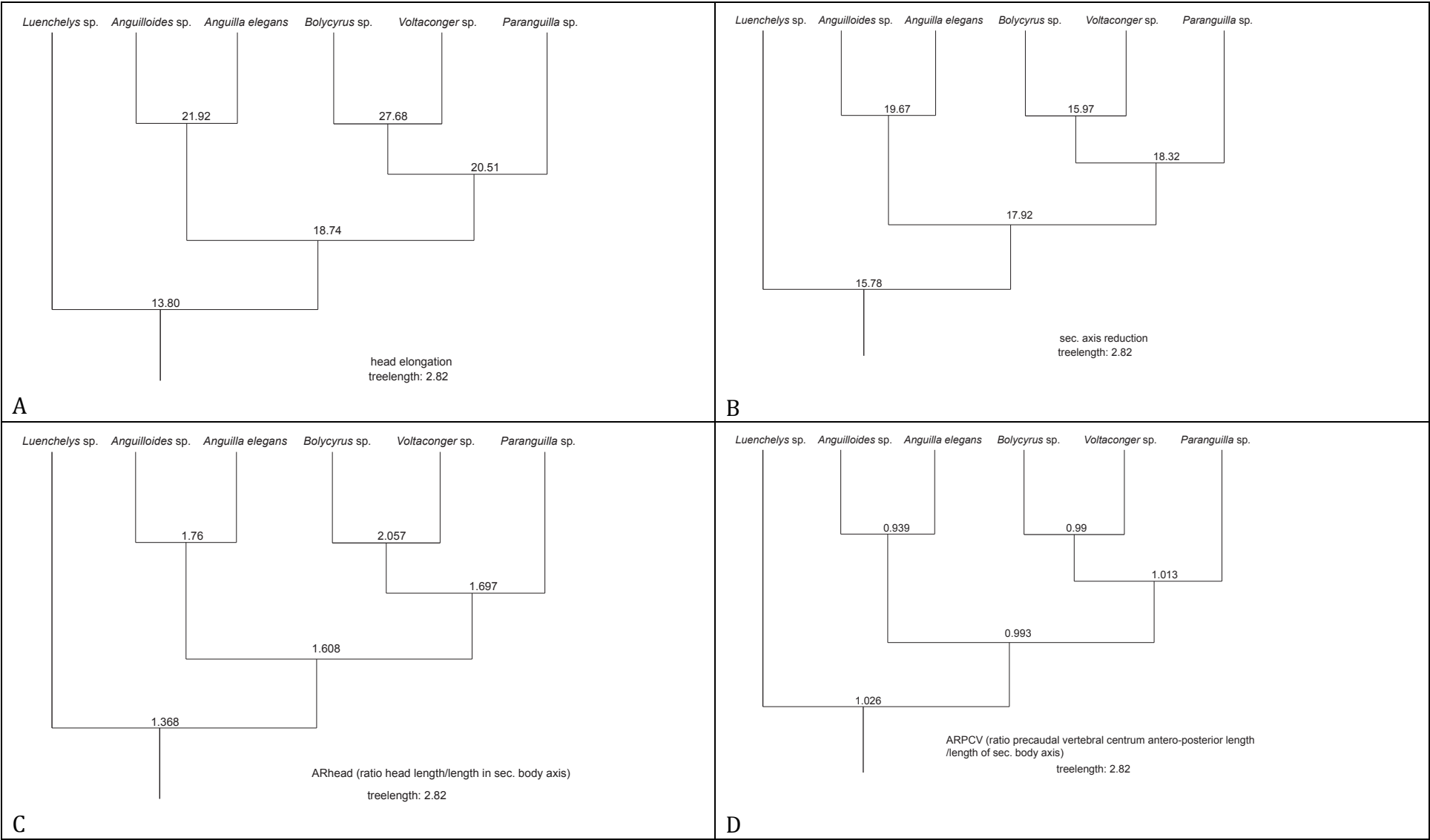

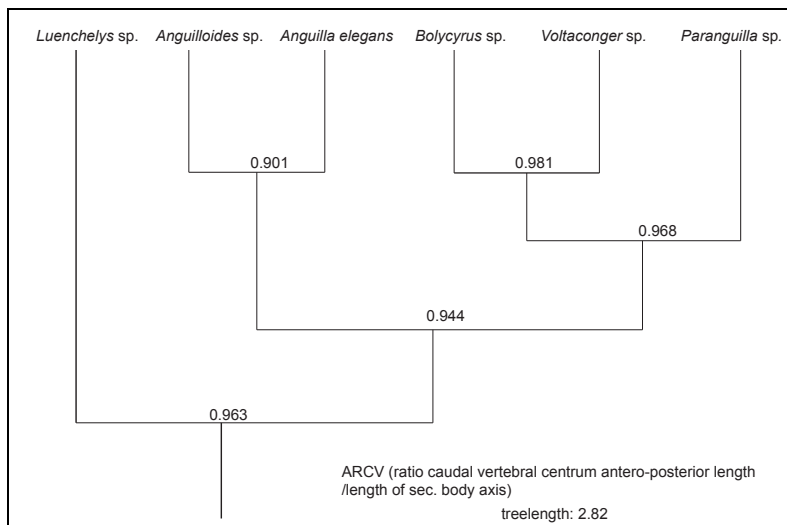

E

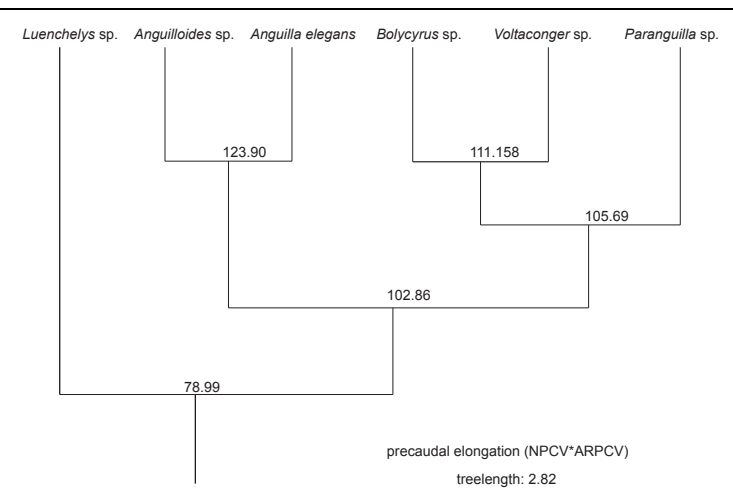

F

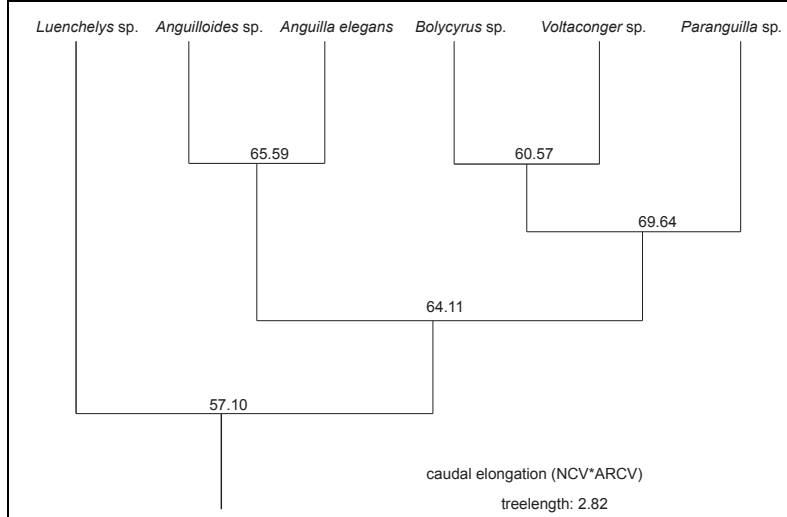

G

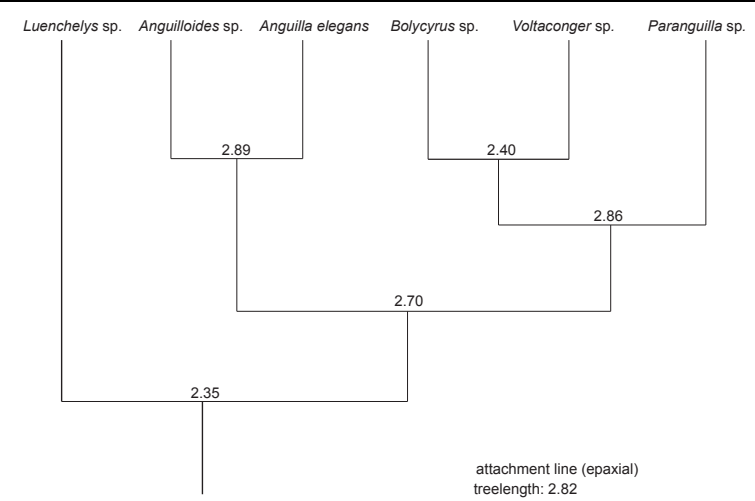

H

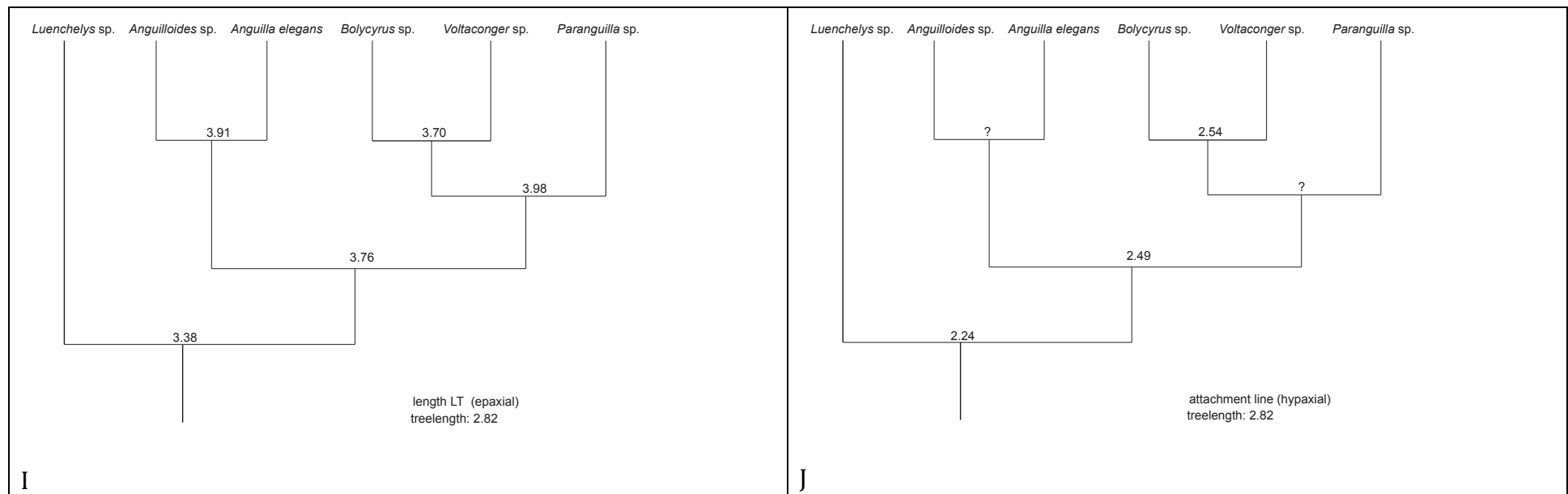

Supplement: Additional file 1: Figure S1. — Reconstruction of ancestral state of distinct parameters of the musculotendinous system and body shape. (PDF 285 kb) [file 12862_2016_728_MOESM1_ESM.pdf]
