# Supplementary material for: Evolution of the locomotory system in eels (Teleostei: Elopomorpha)
Source: BMC Evol Biol. 2016 Aug 11;16:159. doi: 10.1186/s12862-016-0728-7 (PMC4981956; doi:10.1186/s12862-016-0728-7)
Supplement: Additional file 3: Note 1. — A. Supplementary information of the investigated specimens. B. Stratigraphic and geographic distribution of analysed fossil taxa. (DOC 26 kb) [file 12862_2016_728_MOESM3_ESM.doc]

**SI_ Note 1**

**A: Details of the investigated specimens**

†ANGUILLOIDIDAE

ANGUILLIDAE

†*Anguilla elegans* Winkler, 1861: NHMUK 42769

CONGRIDAE

†*Bolcyrus formosissimus* (Eastman, 1905): NMW A.3324 (1853XXVII9)

†*Bolcyrus formosissimus* (Eastman, 1905): MSNVR B.58

†*Bolcyrus formosissimus* (Eastman, 1905): MSNVR T.442

†*Bolcyrus formosissimus* (Eastman, 1905): MSNVR T.467

†*Bolcyrus formosissimus* (Eastman, 1905): MSNVR T.468

†*Bolcyrus formosissimus* (Eastman, 1905): NHMUK P.10428.a

†*Voltaconger latispinus* (Agassiz, 1835): MSNVR VII.A.22

†*Voltaconger latispinus* (Agassiz, 1835): NHMUK P.1889

†*Voltaconger latispinus* (Agassiz, 1835): NHMUK P.17024

†*Voltaconger latispinus* (Agassiz, 1835): MCZ.5346

†PARANAGUILLIDAE

†*Paranguilla tigrina* (Agassiz, 1835): MSNVR T.76,

†*Paranguilla tigrina* (Agassiz, 1835): MSNVR T.990

†ANGUILLOIDIDAE inc. sed.

†*Anguilloides branchiostegalis* (Eastman, 1905): NMW A.3319 (1853XXVII3)

†*Anguilloides branchiostegalis* (Eastman, 1905): MSNVR VII.A.18

†*Anguilloides branchiostegalis* (Eastman, 1905): MSNVR T.887

†*Anguilloides branchiostegalis* (Eastman, 1905): NHMUK P.3876

ANGUILLIFORMES inc. sedis

†*Luenchelys minimus* Belouze et al., 2003a: NHMUK P.62692.a (holotype)

**B. Stratigraphic and geographic distribution of analysed fossil taxa.**

†*Anguilla elegans*: Late Miocene; Oeningen, South Germany

†*Anguilloides branchiostegalis*: Late early Eocene, Ypresian, *Alveolina dainelli* Zone; Pesciara cave site, Monte Bolca, Italy

†*Bolcyrus formosissimus*: Late early Eocene, Ypresian, *Alveolina dainelli* Zone; Pesciara cave site, Monte Bolca, Italy

†*Luenchelys minimus* Belouze et al. 2003a: Sannine Limestone Fm., lower Cenomanian, Late Cretaceous; Namoura, Lebanon

†*Paranguilla tigrina*: Late early Eocene, Ypresian, *Alveolina dainelli* Zone; Pesciara cave site, Monte Bolca, Italy

†*Voltaconger latispinus*: Late early Eocene, Ypresian, *Alveolina dainelli* Zone; Pesciara cave site, Monte Bolca, Italy
